# Shock Properties Characterization of Dielectric Materials Using Millimeter-Wave Interferometry and Convolutional Neural Networks

**DOI:** 10.3390/s23104835

**Published:** 2023-05-17

**Authors:** Jérémi Mapas, Alexandre Lefrançois, Hervé Aubert, Sacha Comte, Yohan Barbarin, Maylis Lavayssière, Benoit Rougier, Alexandre Dore

**Affiliations:** 1 CEA-DAM, GRAMAT, BP80200, F-46500 Gramat, France; 2 CNRS-LAAS, Toulouse University, 7 Avenue du Colonel Roche, BP54200, F-31031 Toulouse, France; aubert@laas.fr (H.A.);

**Keywords:** convolutional neural network, shock properties, mm-wave interferometry, metrology, shock velocity, particle velocity, shock permittivity, shock refractive index

## Abstract

In this paper, a neural network approach is applied for solving an electromagnetic inverse problem involving solid dielectric materials subjected to shock impacts and interrogated by a millimeter-wave interferometer. Under mechanical impact, a shock wave is generated in the material and modifies the refractive index. It was recently demonstrated that the shock wavefront velocity and the particle velocity as well as the modified index in a shocked material can be remotely derived from measuring two characteristic Doppler frequencies in the waveform delivered by a millimeter-wave interferometer. We show here that a more accurate estimation of the shock wavefront and particle velocities can be obtained from training an appropriate convolutional neural network, especially in the important case of short-duration waveforms of few microseconds.

## 1. Introduction

**Figure 1:**
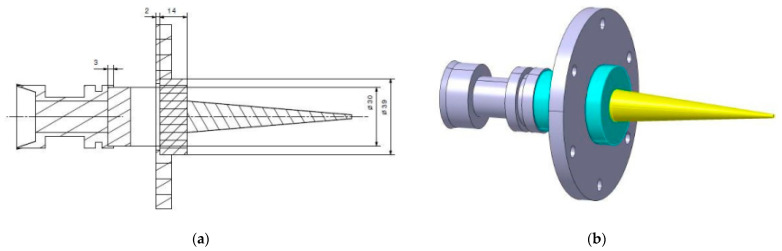
(**a**) Detail of an impact experimental setup to estimate the shock wavefront and particle velocities in dielectric materials using a 94 GHz interferometer (from left to right: projectile, impactor, target support, target and Teflon cone wave guide end), and (**b**) 3D view of a symmetrical impact with the same material as impactor and target in light blue (Teflon cone in yellow).

The physical understanding and modelling of the shock wave propagation in solids has many applications in defense, aeronautics, space and civil areas. In order to simulate the behavior of solids subjected to an impact, it is mandatory to know the mechanical and thermodynamic properties of the pristine and shocked materials. According to the well-documented theory (see, e.g., [1]), the shock wave in dielectric material acts as a moving dielectric interface (or boundary) that propagates faster than the sound in the shocked solid. In the region behind this interface, called the shocked medium, the wave modifies the refractive index of the solid at rest. Moreover, the mechanical impact gives motion to the material, and consequently, it creates a discontinuity in the velocity profile. The velocity of the shocked medium is called the particle velocity, and the fundamental relationship between the shock wavefront velocity *V*_1_ and the particle velocity *V*_2_ is called the shock polar of the material, which can be approximated as follows [2]:

*V*_1_ = *C*_0_ + *s*·*V*_2_ (1)

where *C*_0_ (m·s^−1^) denotes the speed of sound in the pristine medium at the reference state (i.e., in the solid at rest) and *s* is a dimensionless constant. The determination of *C*_0_ and *s* has been the subject of many studies (see, e.g., [2]) and is usually performed from the measurement of *V*_1_ and *V*_2_ [3,4] by using research guns with light gas or powder [5], laser shock [6] or explosives set-ups [7]. Non-invasive techniques were reported to remotely and simultaneously derive the shock wave velocity *V*_1_, the particle velocity *V*_2_ and possibly the refractive index *N*_2_ of the shocked medium from the measurement of two Doppler frequencies in the waveform delivered by a radiofrequency interferometer [8,9,10,11,12,13]. The Doppler effect is a phenomenon where the perceived frequency of sound, light waves or electromagnetic waves changes depending on the relative motion of the source and observer. The impact of the projectile on the material target induces a shock wave. The shock induces a discontinuity change of the material thermodynamic properties before and after the shock front (pressure, velocity, temperature and density). When the interrogation electromagnetic wave passes through the test material, it encounters a moving interface, the shock wave, which is reflective because of the difference in refractive index on either side of the shock wave. The electromagnetic wave is also reflected at the back face of the target, which moves at the speed *V*_2_. These two reflections induce two frequency Doppler shifts in the final signal corresponding, respectively, to *V*_1_ and *V*_2_. Therefore, the Doppler effect is coupled on the amplitude and phase of the interrogation radiofrequency signal and is related to the moving interfaces, and consequently, to the velocities *V*_1_ and *V*_2_. The method in [8] consists of detecting the two Doppler frequencies in the waveform by a fitting process using the linear combination of two sine functions. This radio science paper [8] demonstrates Equations (35)–(37) of this reference. The main conclusion is that there is an analytical solution given by these equations with the three following hypotheses of the physical model: shock velocity *V*_1_ and particle velocity *V*_2_ constant and no dielectric loss. Figure 1 shows a symmetrical impact configuration to study shock properties. The Teflon dielectric waveguide has a variable length ranging from 2 m to 5 m according to the needs, and is used for guiding the millimeter waves to the dielectric sample under shock. The 16 mm in diameter and 80 mm long Teflon cone is glued at the back side of the target to ensure the transition between the dielectric waveguide and the dielectric sample surface. The dielectric cone transition allows the delivery of the millimeter wave generated by the interferometer into the sample during a mechanical impact and to collect the electromagnetic waves reflected by the shocked medium and the surface of the metallic impactor. The impactor is propelled by the Pyrene gas gun and creates a shock at the front surface of the sample, opposite to the dielectric cone interface. The shock physic experiments are plane impact with very limited tilt, less than 5 mrad, performed generally with a light gas gun or a powder gun. So, we analyze the signal in 1D plane configuration without 2D effects from the side release waves. The sustained shock duration is less than 5 µs. The reflectivity of the shock front is about 2% of the input signal, then if the shock front is too low, the detection of the reflected signal could be an issue, depending on the device sensitivity. The noise could be reduced by choosing the lowest oscilloscope caliber in order to avoid signal saturation. We have observed that, for waveforms of sufficiently long duration (>5 µs), the detection and accurate estimation of two Doppler frequencies from the two sinus functions’ fitting technique are possible (see, e.g., [9,10] for the investigation of shocked PolyMethylMetAcrylate dielectrics and TriAminoTrinitroBenzene solids, respectively). However, as the waveform duration decreases and is only of a few microseconds, even less than the pulsation period, lower Doppler frequencies may not be accurately estimated, and consequently, the wavefront and particle velocities *V*_1_ and *V*_2_ cannot be derived from the fitting technique reported in [8]. Short-duration waveforms occur in many circumstances, for instance, when the waveform to be processed is less than a time period long due to dielectric losses. Therefore, it is crucial to significantly extend the applicability of millimeter-wave interferometry to the analysis of very short-duration waveforms of a few microseconds. An Artificial Neural Network technique is proposed in this paper to derive *V*_1_, *V*_2_ and the refractive index *N*_2_ as output neurons of shocked media from time-domain samples of waveforms as input neurons delivered by a millimeter-wave interferometer. The source frequency of the radio-interferometer is 94 GHz, and the wavelength is 3.2 mm. The sample rate is 1 Gsamples/s, and the signal duration is generally a few microseconds. The interrogation distance is between a few millimeters to a hundred millimeters in the target material. Low noise electronic components, especially the IQ mixer, are used to build the radiofrequency interferometer in order to ensure better accuracy. The accuracy on the shock physic velocities depends on the uncertainty of the dielectric properties of the material.

Fully dense Neural Network (NN) models were applied in [14,15,16,17] to extract frequencies of interest from some waveforms, but these models are not suitable here. As they require a fixed number of input neurons, these models cannot process waveforms with a variable number of samples. An alternative NN technique consists of using Convolutional Neural Network (CNN) models (see, e.g., [18,19]). CNNs have been widely used in the field of speech recognition, acoustic analysis for leakage, and electrocardiogram analysis for long duration signals of more than one millisecond. The first approach is to use Fast Fourier Transform (FFT), when the frequency is constant, and/or the wavelet method, if the frequency varies. CNN is generally applied for spectrograms to identify complex features and noisy environments are taken into account. The large possible variations in the image or speech recognition and 2D or 1D signals are overcome by the architecture of CNN, thanks to the easier process of weight configurations during training and thanks to local correlations, in order to extract local features [20].

However, in the case of short signals obtained by ultrafast sensors (less than 10 µs), there is no conclusive scientific literature on the use of CNNs. In the case of characterizing the dielectric properties of energetic materials, there is no previous work including the use of CNNs.

The raw signal that we would like to analyze has two frequencies, but they are sustained only during less than a time period. The classical frequential analyses (Fourier Transform, wavelet, etc.) are inaccurate in this case, because there are not enough time periods to give a conclusive result.

**Figure 2:**
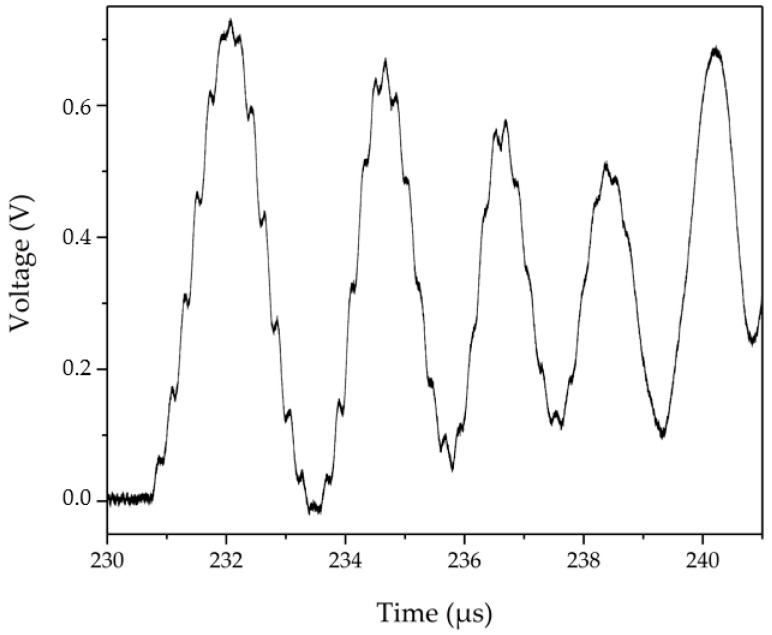
Typical waveform delivered by a 94 GHz interferometer during impact experiment on a TriAminoTrinitroBenzene (TATB) material (refractive index *N*_1_ is of 1.78). The derivation of Doppler frequencies from the fitting process gives the following estimation: *N*_2_ = 2.37 (shocked medium refractive index), *V*_1_ = 3850 m·s^−1^ (shock wavefront velocity) and *V*_2_ = 385 m·s^−1^ (particle velocity).

We show in this paper that the accurate estimation of velocity *V*_1_ of the shock wavefront, the particle velocity *V*_2_ and the refractive index *N*_2_ of the shocked medium can be estimated from a dedicated CNN, especially for waveforms less than a pulsation period. Indeed, only the first two microseconds are analyzed from the typical waveform, presented in Figure 2. The shock time arrival is near 230.8 µs, when the raw signal oscillation starts. The signal is analyzed from 230.8 µs to 233.5 µs. Between 233.5 µs and 239 µs, the variations of the peak-to-peak signal amplitude are associated with the dielectric loss of the material. After 239 µs, the shock wave enters in the Teflon cone. The high frequency disappears because the shock wave pressure is too low. The period 1/f1 is associated with high frequency, around 10 periods between the shock arrival (230.8 µs) and 233.5 µs. The period 1/f2 is associated with the low frequency, first period between the shock arrival (230.8 µs) and 233.5 µs. The sample is glued on a metallic transfer plate, which is why, in this case, the radiofrequency interferometer does not show the impactor reflection.

The manuscript is organized as follows. Section 2 briefly describes the previously reported method used to derive the shock wavefront and particle velocities from the estimation of two Doppler Frequencies, and discusses the limitations of this method. Section 3 describes the development of a new method based on Convolutional Neural Networks to overcome the limitations of the previously reported method. In Section 4, the performances of the two methods are compared for the derivation of the shock wavefront and particle velocities from the same set of measured waveforms.

## 2. Shock Wavefront and Particle Velocities Derived from Doppler Frequencies

The shock wavefront in a dielectric material is usually modeled by a moving interface (see, e.g., [21]), which separates the material in two dielectric regions; the region in front of the interface is called the *pristine medium* with a refractive index *N*_1_, while the region behind the interface is called the *shocked medium* with a refractive index *N*_2_. The incident electromagnetic field is then subjected to the reflection by and transmission through the dielectric interface with a Doppler frequency shift. In addition, the electromagnetic field encounters losses in the material. Two configurations are analyzed throughout the paper, the single and double interface configurations.

In the so-called *single interface configuration*, the dielectric interface models the shock wavefront. It moves toward a millimeter-wave interferometer, and the velocity *V*_1_ of the interface is derived from the extraction of the Doppler frequency shift in the waveform delivered by the interferometer. The extraction requires the prior knowledge of pristine medium refractive index *N*_1_. The single interface configuration offers an exact solution for the waveform [8], which is used here to investigate the eventual benefits of the Neural Network approach for estimating the shock wavefront velocity *V*_1_.

**Figure 3:**
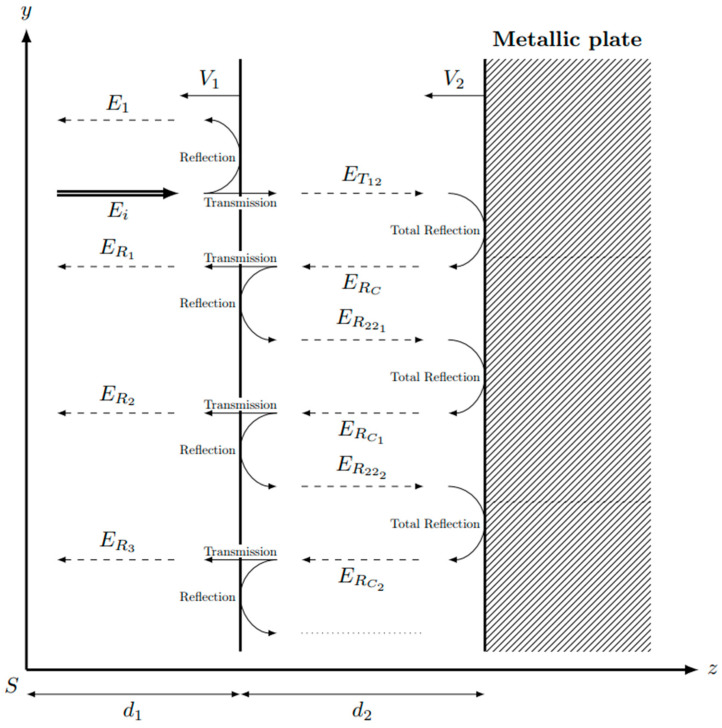
Scheme of the double interface configuration. The incident electric field *E_i_* is normal to the shock wavefront, that is, the moving interface between the pristine material and shocked medium, with *E*_1_, first reflection; *E*_R i_, following transmission/reflection/transmission; *E*_T_, first transmission; *E*_Rc i_, reflections on the metallic plate. The pristine material thickness is d_1_. The shocked medium thickness is d_2_.

The *double interface configuration* takes into account the metallic impactor or the transfer plate. Therefore, in addition to the reflection by the (*E*_R_) and the transmission through the shock wave interface (*E*_T_), the electromagnetic field transmitted by the interferometer experiences the reflection by and transmission through the moving interface between the shocked medium and the metallic plate (see Figure 3). In this study, the metallic plate is assumed to be perfectly conductive, and consequently, the total electromagnetic reflection occurs at its surface, called *E*_Rc_. Following [8], both the velocity *V*_1_ of the shock wavefront and the velocity *V*_2_ of the metallic interface between the impactor (metallic plate) and the shocked medium may be derived from the measurement of two Doppler frequencies in the reflected electric field *E_R_ = E_R_*_1_ *+ E_R_*_2_ *+ E_R_*_3_ *+* ….

As illustrated in Figure 2, typical measured waveforms actually exhibit two oscillations, whose frequency and magnitude are used here to estimate the velocities *V*_1_ and *V*_2_ (it is assumed here that the refractive index *N*_1_ of the studied pristine material is known). This estimation may be performed from a fitting process which approximates the waveform by the linear combination *s*(*t*) of two sine functions given by the following:

*s*(*t*) = *A*_1_·*sin(2*π*f*_1_·*t* + *φ*_1_) *+ A*_2_·*sin(2πf*_2_·*t + φ*_2_) (2)

where the fitting parameters are *f*_1_ and *f*_2_ (i.e., the unknown Doppler frequencies), *A*_1_, *A*_2_, *φ*_1_ and *φ*_2_. We have observed that, for waveforms of sufficiently long duration (>5 µs), the fitting process allows for the detection of the two Doppler frequencies of interest, but as the waveform duration decreases, the accuracy of the lowest frequency estimation degrades gradually, and as a result, velocities *V*_1_ and *V*_2_ cannot be estimated precisely.

## 3. Shock Wavefront and Particle Velocities Derived from CNN Approach

The proposed approach requires the careful selection of the triplets (*V*_1_, *V*_2_, *N*_2_) that are used in the simplified electromagnetic model of moving dielectric interface(s) reported in [8] for computing the waveforms and training the CNN.

**Figure 4:**
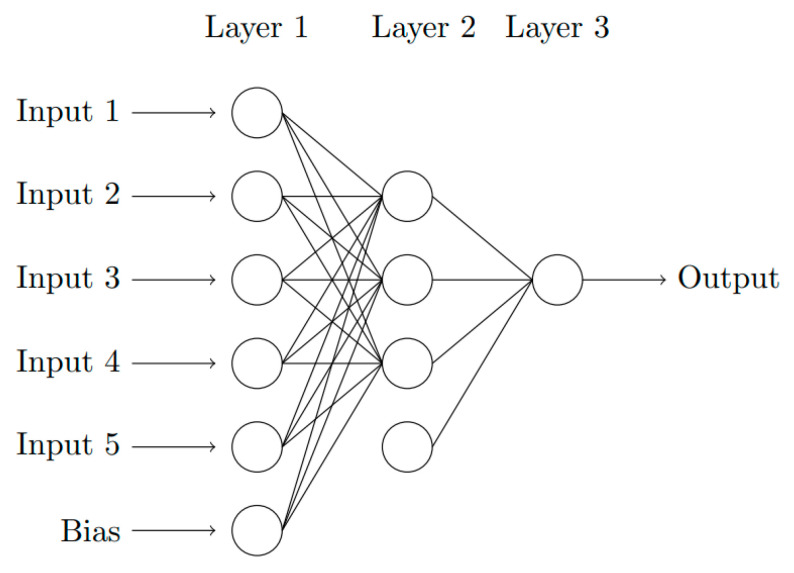
Simple architecture of a Neural Network (The circles represent the nodes).

An Artificial Neural Network (NN) with Multi-Layer Perceptron (MLP) [14] is an assembly of layers, each composed of several neurons, or nodes. As a biological neuron, each neuron linearly combines the outputs of the previous layer and applies an activation function to obtain the output. This function is often non-linear. Once the architecture of the NN is chosen, the learning process is launched. This stage consists of adjusting the parameters of each node to fit on a combination of inputs and outputs of the network. Here, the input is the *N* samples of the time-domain waveform delivered by the millimeter-wave interferometer during an impact experiment on dielectric materials, and the single output is the velocity *V*_1_ of the shock wavefront, or the particle velocity *V*_2_, or else the refractive index *N*_2_ of the shocked medium. Figure 4 shows an example of such NN. It consists of three layers; the first layer has five inputs and one bias, the second layer has three neurons and one bias and the third layer is composed of one output neuron which provides the estimation of the quantity of interest (that is, *V*_1_, *V*_2_ or *N*_2_). The bias is used to influence the output without interfering with the weights and the inputs. In the second layer, the output of each node is computed as follows using relation (3).

*Output = f(w*_1_ × *Input*_1_ + *w*_2_ × *Input*_2_ + *w*_3_ × *Input*_3_ + *w*_4_ × *Input*_4_ + *w*_5_ × *Input*_5_ + *w_b_ × b)* (3)

where *f* is the scoring function, *b* denotes the bias, *w_b_* designates the weight of the bias, *Input_i_* are the inputs *i* and *w_i_* is the weight of *Input_i_*.

In our investigation, the measurement data used in the input layer of the NN are annotated, i.e., they are known before they are processed in the NN. This case is also known as “supervised learning”, which is generally used to sort two types of problems as follows:

- Regression problems, where the problem is to estimate a quantity variable (*V*_1_, *V*_2_ or *N*_2_ in our study);
- Classification problems, where the problem is to predict a qualitative variable (i.e., a state, a category, etc.).

For any architecture, it would be possible here to process the waveform as a regression or classification problem, because it would either be possible to obtain the accurate value of a velocity as output after the waveform processing, or to determine a range in which the velocity may be included. Anyway, for reasons of diagnostic accuracy, the analysis of waveforms will be considered here as a regression problem.

In view of the state-of-the-art (see, e.g., [15,16]), a fully connected dense model—i.e., each neuron in a layer receives an input from all the neurons of the previous layer—can accurately estimate the spectral content of some waveforms. The major drawback of this model is that the number of input neurons is fixed, and consequently, the same NN cannot process the variable number of waveform samples. Another limitation is that NN-based spectral analysis is not automated, so the signal processing is applicable to specific waveforms. Therefore, a fully connected dense model is not suitable to solve our inverse problem, either from using regression or classification methods. Another approach, based on a Convolutional Neural Network (CNN), is implemented here for the two configurations described in Section 2. The following is a well-known technique in raw signal processing [18,19]: The convolutional layers are filters that extract patterns from the signal; then, these patterns are processed by the dense layers to fit the output. The CNN is implemented here using Python 3.7.6 with the module Keras 2.8.0 [22] and the backend TensorFlow 2.9.3 [23]. The number of convolutional and dense layers is computed by fitting various architectures on the validation waveform data. The architecture of the network is identical for the following two configurations:

- In the *single medium configuration*, two networks are studied; the first network has the shock wavefront velocity *V*_1_ as single output and the second has only the refractive index *N*_2_ of the shocked medium as output;
- For the *double medium configuration*, three networks are studied; the first network has the shock wavefront velocity *V*_1_ as output, the second one has the particle velocity *V*_2_ and the third one has the refractive index *N*_2_ of the shocked medium.

**Figure 5:**
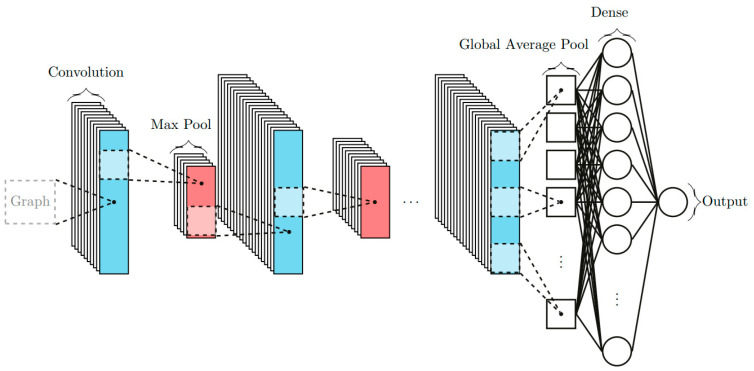
Scheme of the CNN used for determining the shock wavefront *V*_1_, the particle velocity *V*_2_ or the refractive index *N*_2_ of the shocked medium from waveforms delivered by the 94 GHz millimeter-wave interferometer.

The waveform samples are normalized from a Glorot normal initialization [24]. This approach was found to be more efficient than a single CNN with two or three outputs. The inputs for the networks are the refractive index *N*_1_ of the pristine material, the operating frequency (94 GHz) of the millimeter-wave interferometer, the time step used for the sampling of waveforms and the samples of waveforms delivered by the interferometer. The first architecture was made with a 16-layer neural network, inspired by the A.Dore Thesis [25,26]. Then, the architecture was enlarged to 18 layers in order to improve the precision and the generalization prediction. For this publication, the train set is 246,000 samples, the validation set is 30,000 samples and the test set is also 30,000 samples. For the comparisons between the DFA and NNA, the number of samples is 1000 for the single medium configuration and for the double medium configuration. The final network has four convolutional layers and seven dense layers (see Table 1). The convolutional layers perform the filtering operation, while the dense layers create linear combinations with bias. The maximum pooling layer selects the maximum value in a range of neurons. Batch normalization is a well-known technique in neural networks to overcome overfitting, which may occur when the algorithm performs well on training data but performs badly on any other data. This regularization technique allows the algorithm to keep its generalization ability [27]. The global average pooling layer makes the average in the selected range and flattens the filters from the previous convolutional layer to decrease the data size for the next layer, and consequently, to reduce the calculation time without interfering with the training process. Figure 5 sketches the action of every layer.

The learning process is performed from the simulated waveforms provided by the electromagnetic model developed in [8]. The model is applied to create waveforms with multiple initial parameters. It allows using larger networks and prevents overfitting, which may occur when a limited number of experimental data is available. Moreover, the main advantage of using simulated inputs for the CNN training is that we can generate a large data set which makes possible the derivation of the accurate estimation of velocities or refractive index. The main idea is that the waveform is composed of two main contributions. The first is the electric field directly reflected by the shock wavefront. The second contribution combines multiple reflections of the electric field inside the dielectric sample. For each of contributions, the reflection and transmission coefficients are computed. The adequate number of reflections in the shocked medium (see Figure 3) is derived from the numerical convergence of the total reflected electric field. Table 2 reports the mean difference between the computed waveforms. The chosen number of reflections in the shocked medium is set to four, as the difference with the waveform with five internal reflections does not exceed 0.004%. The learning process is performed with the Adam optimizer with the coefficients given in [28] and with a mean squared error loss. The maximum number of full training cycles or *epochs* is set to 200. However, the learning rate decreases if the loss is constant over five epochs. In practice, the maximum number was never reached, as the loss converged rapidly. To ensure that the loss is not on a plateau, ten more epochs are computed after reaching numerical convergence. To avoid overfitting, new validation data are computed at each epoch.

The refractive index of both the pristine material and the shocked material, denoted, respectively, by *N*_1_ and *N*_2_, and the velocity *V*_1_ of the shock wavefront and the particle velocities *V*_2_ are randomly generated for each waveform. The time duration of waveforms is also randomly modified to account for various experimental conditions. The quantities of interest are normalized before the validation step during the learning phase. The boundaries for each parameter are listed in Table 3. These bounds are chosen to be representative of the experimental values. Following [29], the refractive index of the dielectric material increases when submitted to a shock wave. Therefore, during the learning stage, *N*_2_ is computed as the summation of *N*_1_ with a random number ranging from 0 to 1. All the signals for the train set, the validation set and the test set are generated with the Table 3 parameter ranges (*N*_1_, *N*_2_, *V*_1_, *V*_2_, measurement duration). Thus, *V*_1_, *V*_2_ and *N*_2_ are the initial parameters chosen to determine the frequencies and the amplitude values and are therefore directly known and correlated to these values. So, it is very helpful for the uncertainty analysis, because the pristine values (*V*_1_, *V*_2_, and *N*_2_) are compared directly to the retrieved ones using the DFA or NNA method. Therefore, the frequencies and the amplitudes are intermediate values. Especially for the DFA, they are the output of the double sinus method, and the associated *V*_1_, *V*_2_ and *N*_2_ are calculated back with the physical model using Equations (35)–(37) in reference [8].

## 4. Results and Discussion

### 4.1. Single Medium Configuration

**Figure 6:**
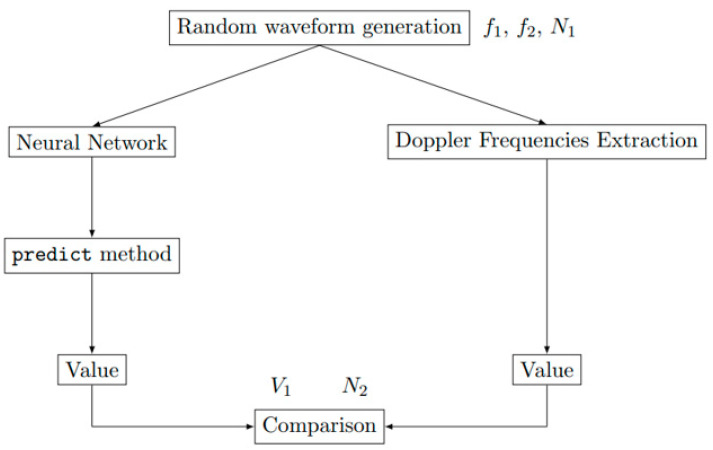
Sketch for the comparison of the Neural Network Approach (see Section 3) and the Doppler Frequency Approach (see Section 2) applied to the single medium configuration.

**Figure 7:**
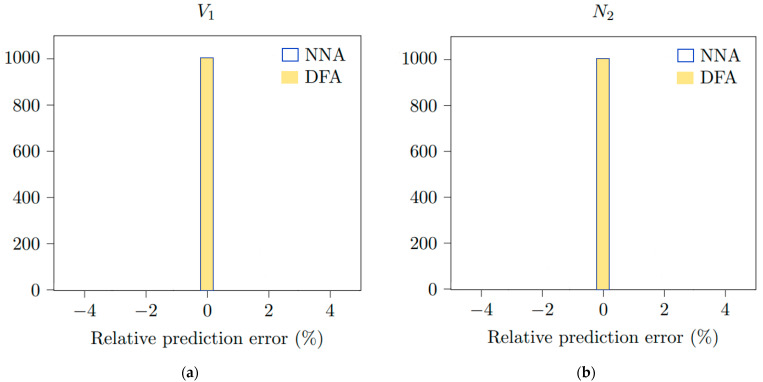
(**a**) Relative prediction error distribution on *V*_1_ derived from NNA and DF, and (**b**) relative prediction error distribution on *N*_2_ determined from NNA and DF (single medium configuration). The number of comparison samples is 1000.

To compare the performances of the two methods reported in Section 2 and Section 3, that is, the technique based on the extraction Doppler frequencies [8] (DFA, which stands for Doppler Frequency Approach) and the proposed Neural Network Approach (NNA), one thousand random waveforms with a length of 10,000 points based on the Table 3 ranges are computed and processed. With the chosen CNN, the output values are obtained from the predict method from the Keras 2.8.0 module [22]. As the fitting process is direct, the value is simply calculated by the Fast Fourier Transform and directly compared with the outputs of the CNN. The procedure is sketched in Figure 6. The number of samples versus the relative prediction error are presented in Figure 7 on *V*_1_ and *N*_2_ for the NNA and DFA. The error displayed on the graph abscissa is the difference between the values used to generate the signal and the values predicted by the NNA or DFA after the analysis of this same signal. No difference is obtained between the DFA and NNA for the single medium configuration. For the shock wavefront velocity *V*_1_ and the refractive index *N*_2_ of the shocked medium, the two methods provide similar results and accuracy. Following these encouraging results, the neural network is applied in Section 4.2 to the double medium configuration, which consists of a much more realistic model for the practical situation.

### 4.2. Double Medium Configuration

**Figure 8:**
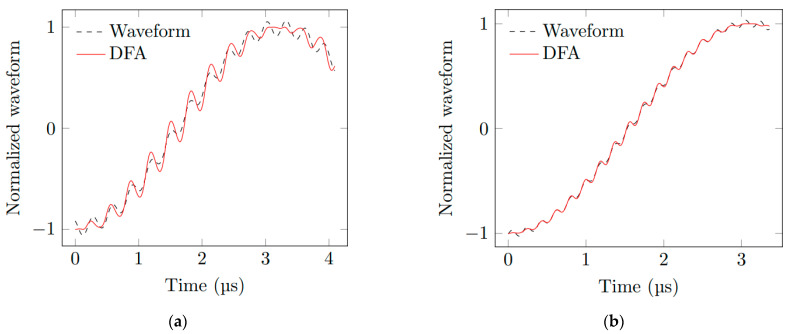
Dimensionless waveforms computed with *V*_1_ = 4000 m s^−1^, *V*_2_ = 400 m s^−1^ and *N*_2_ = 2 (dashed line), and the waveforms derived from the DFA fitting process (in red line) for various correlation coefficients, (**a**) $\rho_{X,Y}$ = 0.997 and (**b**) $\rho_{X,Y}$ = 0.9998.

**Figure 9:**
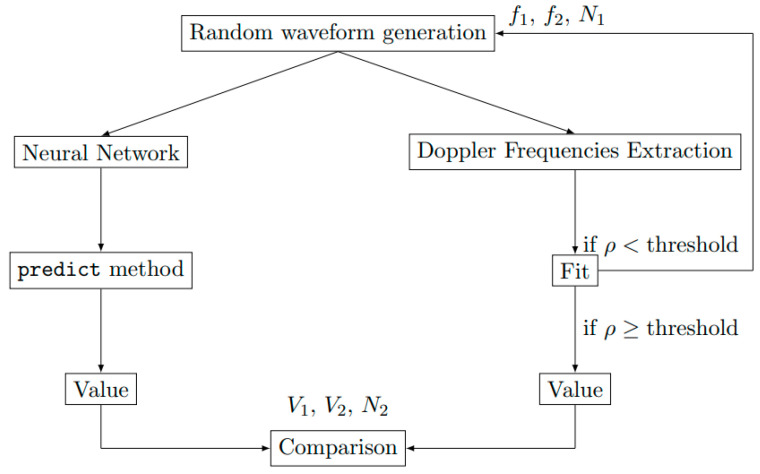
Sketch for the comparison of the Neural Network Approach (see Section 3) and the Doppler Frequency Approach (see Section 2) applied to the double medium configuration.

**Figure 10:**
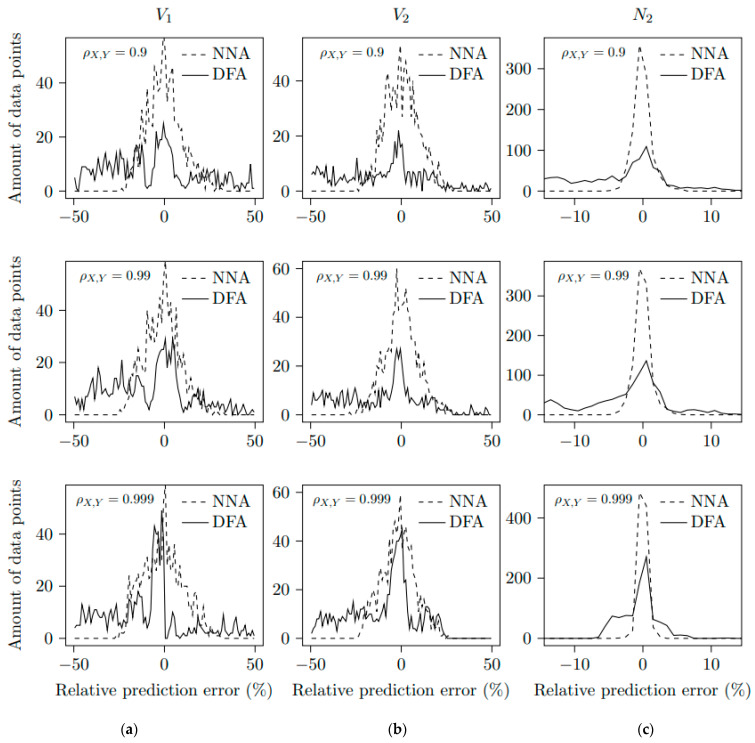
Relative prediction error distribution given by NNA and DFA with thresholds $\rho_{X,Y}$ = 0.9 (up), 0.99 (middle) and 0.999 (down) for determination of (**a**) the shock wavefront velocity *V*_1_, (**b**) particle velocity *V*_2_ and (**c**) the refraction index *N*_2_ of the shocked medium.

For the double medium configuration, one thousand random waveforms with a length of 10,000 points based on the Table 3 ranges are computed from the electromagnetic model reported in [8] and are used for training the CNN. As in Section 4.1, the output values are obtained by using the predict method from the Keras 2.8.0 module. For deriving the velocity *V*_1_ or *V*_2_ from the DFA, the resolution is not straightforward; as the number of comparison points increases, the fitting process using two sine functions (see Section 2) must be performed automatically by using an initial guess vector for the parameters. The fitted waveform is computed and correlated with the input waveform. Let the correlation coefficient $\rho_{X,Y}$ of two investigated waveforms *X* and *Y* be defined as follows:

(4) $$\rho_{X,Y}=\frac{cov\left(X,Y\right)}{\sigma_{X}\sigma_{Y}}$$

where *cov* denotes the co-variance operator, while $\sigma_{X}$ and $\sigma_{Y}$ designate the standard deviation of *X* and *Y*. If $\rho_{X,Y}$ is smaller than a prescribed threshold, the input waveform is discarded because the fitting process is considered to be unsuccessful. This method is repeated until 1000 waveform samples with a length of 10,000 points have been obtained. Two examples of computed waveforms are plotted in Figure 8, as well as the result of the DFA and corresponding correlation coefficients. The procedure is sketched in Figure 9. For comparison purposes, we determine the difference between *V*_1_, *V*_2_ and *N*_2_ estimated from the DFA and NNA, with the exact values selected for the waveform computation. The mean value *M* of the difference and the standard deviation for each method are reported in Table 4. A systematic error (i.e., the mean is not zero) is present for both methods, but it is smaller for the NNA than for the DFA. The NNA also yields a smaller standard deviation than the DFA. As expected, the higher the correlation coefficient $\rho_{X,Y}$ (or equivalently, the more accurate the derivation of Doppler frequencies and magnitudes), the more accurate the estimation of *V*_1_, *V*_2_ and *N*_2_ provided by the DFA. However, as the correlation coefficient increases, the systematic error on the estimation of *V*_1_, *V*_2_ and *N*_2_ provided by the NNA is constant, even for short-duration waveforms. The number of samples versus the relative prediction error are presented in Figure 10 for the DFA and NNA. Theses distributions are displayed for three different correlation coefficients, 0.9, 0.99 and 0.999. The relative prediction error is the difference between the predicted values and the true values of *V*_1_, *V*_2_ and *N*_2_. Table 4 is the analyzed table of Figure 10. The mean value and the standard deviation for CNN are, respectively, less than 1.1% and 11.4% for the parameter *V*_1_. The mean value and the standard deviation for CNN are, respectively, less than 1.6% and 9.4% for the parameter *V*_2_. The mean value and the standard deviation for CNN are, respectively, less than 0.1% and 0.1% for the parameter *N*_2_. So, compared to the DFA in Table 4 and Figure 10, the mean values of the NNA test set are near zero, showing a more precise method as a centered distribution, and the standard deviations of the NNA test set are reduced, showing less dispersed values. For the very large correlation coefficient, the methods of the NNA and DFA are quite similar. This seems logical, because the DFA is more precise in this case. However, we continue to observe large deviations for the DFA method. As typical experimental values for the correlation coefficient are between 0.9 and 0.99 [10], the NNA should then be preferred when processing short-duration waveforms, especially for estimating the particle velocity *V*_2_ and refractive index *N*_2_ of the shocked medium.

## 5. Conclusions

In this paper, a convolutional neural network is proposed for solving an electromagnetic inverse problem involving solid dielectric materials subjected to mechanical impacts. From the computed waveforms delivered by a millimeter-wave interferometer, the convolutional network provides more accurate estimations of the shock wavefront velocity in the shocked materials, the particle velocity, as well as the refractive index of the shocked medium, for short-duration waveforms of a few microseconds less than a time period long, compared to the Doppler Frequency Analysis, based on the fitting parameters of the sum of two sine functions. These results significantly extend the application of millimeter-wave interferometry to the investigation of dielectric materials subjected to steady shocks. Work is ongoing to improve the network architecture in order to reduce the standard error prediction for each parameter.

## Figures and Tables

**Table 1:** Final CNN for each parameter *V*_1_, *V*_2_ and *N*_2_ as output.

Index of Layer	Type of Layer	Keras Name	Activation Function	Properties
1	Convolution	Conv1D	Rectified Linear Unit (ReLU)	72 filters, length 10
2	Normalization	BatchNormalization		
3	Convolution	Conv1D	ReLU	144 filters, length 10
4	Normalization	BatchNormalization		
5	Pooling	MaxPooling1D		
6	Convolution	Conv1D	ReLU	288 filters, length 10
7	Normalization	BatchNormalization		
8	Pooling	MaxPooling1D		
9	Convolution	Conv1D	ReLU	576 filters, length 10
10	Normalization	BatchNormalization		
11	Pooling	GlobalAveragePooling1D		
12	Dense	Dense	ReLU	50 neurons
13	Dense	Dense	tanh	60 neurons
14	Dense	Dense	tanh	40 neurons
15	Dense	Dense	tanh	30 neurons
16	Dense	Dense	ReLU	20 neurons
17	Dense	Dense	tanh	10 neurons
18	Dense	Dense	hard sigmoid	1 neuron

**Table 2:** Mean difference of the total reflection between signals for different number of internal reflections in the shocked dielectric sample.

Number of Considered Reflections in the Shocked Medium	Total Reflection Mean Difference (%)
1 and 2	5.7
2 and 3	0.5
3 and 4	0.05
4 and 5	0.004
5 and 6	0.0004

**Table 3:** Parameters boundaries for the learning step of the CNN.

Parameter	Minimum Value	Maximum Value
Material at rest refractive index *N*_1_	1	2
Shocked material refractive index *N*_2_	1	3
Particle velocity *V*_2_ (m s^−1^)	300	500
Shock wavefront velocity *V*_1_ (m s^−1^)	3000	5000
Measurement duration (µs)	2	3.5

**Table 4:** Mean and standard deviation of the difference between *V*_1_, *V*_2_ and *N*_2_ estimated from DFA and NNA, with the exact values selected for the waveform computation.

Method Name	*V*_1_		*V*_2_		*N*_2_		$\rho_{X,Y}$
	*M* (%)	*σ* (%)	*M* (%)	*σ* (%)	*M* (%)	*σ* (%)	
Neural Network Approach	−0.1	9.8	0.6	9.4	−0.1	−0.1	0.9
Doppler Frequency Approach	−5.9	38.1	37.9	115.2	1.4	1.4	
Neural Network Approach	−1.1	10.0	0.1	9.2	−0.1	−0.1	0.99
Doppler Frequency Approach	−9.6	29.8	32.2	100.7	7.0	7.0	
Neural Network Approach	−1.0	11.4	−1.6	9.3	0.0	0.0	0.999
Doppler Frequency Approach	−7.6	33.3	−6.5	35.9	2.6	2.6	

## Data Availability

The data presented in this study are available on request from the corresponding author.
